# Zeolitic imidazolate framework-8/polyaniline nanocomposite-based electrochemical sensor for sensitive detection of imidaclothiz

**DOI:** 10.2116/analsci.21P063

**Published:** 2023-10-25

**Authors:** Ziyan He, Zhihui Li, Tao Feng, Jin Cui, Fengting Li

**Affiliations:** 1 College of Environmental Science and Engineering, Tongji University, Shanghai, 200092 China; 2 Xintai Water Treatment Technology Co. LTD, Zaozhuang, 277000 Shandong China

**Keywords:** ZIF-8, Polyaniline, Electrochemical sensor, Neonicotinoids, Square wave voltammetry

## Abstract

Imidaclothiz (IMZ) is a class of neonicotinoid insecticide which can pose potential threat to human health and be frequently detected in water and foods. Herein, a zeolitic imidazolate framework-8/polyaniline (ZIF-8/PANI) nanocomposite has been modified on the surface of glassy carbon electrode (GCE) for the electrochemical determination of IMZ, and the electrochemical detection performance of the modified electrode was investigated by cyclic voltammetry (CV) and square wave voltammetry (SWV). With the large surface area of ZIF-8 and great electric conductivity of PANI, the ZIF-8/PANI-modified electrode showed a high catalytic performance towards IMZ reduction in PBS. Under the optimized conditions, the linear range was from 1.0 × 10^−7^ to 1.0 × 10^−5^ mol/L and the limit of detection was as low as 2.5 × 10^−8^ mol/L (S/N = 3). In addition, the developed sensor displayed high reproducibility, excellent stability, and applicability in real vegetable sample analysis, indicating that the proposed method offered an alternative approach for IMZ residues analysis.

**Graphical abstract:**

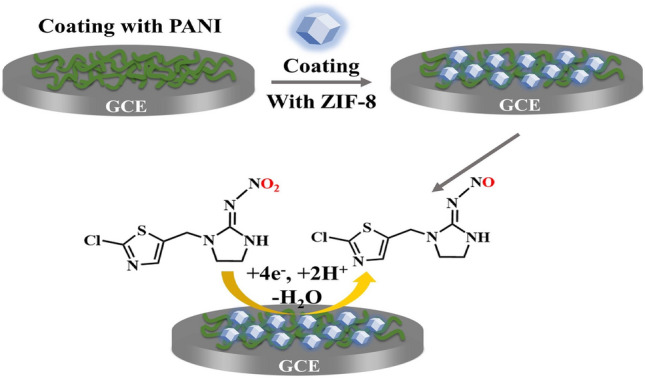

**Supplementary Information:**

The online version contains supplementary material available at 10.2116/analsci.21P063.

## Introduction

Imidaclothiz (IMZ) is a synthetic neonicotinoid insecticide, which has been growing fast since introduced in the mid of 1990s. Because of the relatively low toxicity to non-target organisms and environment compared to other insecticides [1], neonicotinoids are commonly used as insect controls in a variety of crops such as corn, cereals, and oilseed rape [2]. However, neonicotinoid residues can be detected frequently in environmental water, and due to their good water solubility, neonicotinoids are easily taken up by the growing plant and distributed to all tissues [3]. Therefore, human beings could be exposed to neonicotinoids through the neonicotinoid-contaminated water and foods, respectively [4, 5]. Several studies have reported that long-term exposure to neonicotinoids can lead to impairment intellectual development for children and pulmonary dysfunction for adults [6–8]. Thus, it is necessary to establish a rapid, convenient, accurate detection method for IMZ residues analysis. So far, some analytical techniques, such as high-performance liquid chromatography (HPLC) [9], HPLC coupled to tandem mass spectrometry (HPLC–MS/MS) [10], and enzyme-linked immunosorbent assay (ELISA) [11, 12] have already been developed to determine IMZ residues. In spite of their strong stability and high sensitivity, the high cost of equipment and the complicated pretreatment of samples limit their widespread use. Owing to the apparent advantages of low cost, ease of operation, rapid response, and high sensitivity, electrochemical sensor has been considered to be a promising method for chemicals detection. For example, Veronika et.al demonstrated the graphene oxide modified glassy carbon electrode for electrochemical detection of two neonicotinoids, thiamethoxam and imidacloprid. The results showed a good linear range of 10–200 µM for both analytes and the detection limits were determined as low as 8.3 µM and 7.9 µM for thiamethoxam and imidacloprid, respectively [13]. However, for environmental safety, it is still necessary to improve the low-level detection of neonicotinoids. Thus, designing excellent electrocatalytic materials for the electrode modification is a challenging approach.

Metal–organic frameworks (MOF) are a kind of porous crystalline materials assembled from metal-based nodes and organic linker [14, 15]. With high surface area, controllable pore size, diverse functionalities, and homogeneously dispersed active sites, MOFs are considered as superior materials for electrochemical sensor [16]. Among the MOFs, zeolitic imidazolate framework-8 (ZIF-8), built from Zn^2+^ ions and 2-methylimidazolate, has attracted extensive attention. In addition to remarkable stability in water, nano-sized ZIF-8 usually has large numbers of exposed active sites and better mass diffusion of the analyte, which can help to improve the electrocatalytic activity and detection sensitivity [17, 18]. There are some electrochemical sensors based on ZIF-8 have been reported to detect heavy metal ions [19], glucose [20], pentachlorophenol [21], etc. Nevertheless, the direct application of ZIF-8 in electrochemical sensor area is usually limited by its poor conductivity [22]. To address this problem, some excellent conductive and electroactive materials are proposed to combine with ZIF-8, further improving the electrochemical performance of the modified electrode. For example, Shi et al. have encapsulated Cu nanoparticles in ZIF-8 and the electrochemical experiments showed that the incorporation of Cu nanoparticles greatly enhanced the stability and electrocatalysis of the ZIF-8 [23]. In addition, Zheng et al. prepared a graphene-ZIF-8 nanocomposite-modified electrode and used it as a highly sensitive and selective sensor for the electrochemical determination of dopamine [24].

Polyaniline (PANI) is a widely used polymer and has been found as modified material for electrochemical sensor with promising features, including relatively high conductivity, excellent chemical and electrochemical stability, easy synthesis, as well as low monomer cost [25, 26]. Furthermore, its functionality can be extended by introducing a secondary component, such as nanomaterials [27]. It has been experimentally shown that the synergistic effect between the individual components can enhance the characteristics of nanocomposite and, thus, expand its application scope [28]. Depending on the secondary component, the nanocomposite can be multi-functionalized, as in the case of PANI/Fe_3_O_4_ nanocomposite which exhibited both conductivity and magnetism [29]. Hence, it’s an effective strategy to construct ZIF-8/PANI nanocomposite-modified electrochemical sensor for the sensitive and rapid detection of IMZ.

In this work, glassy carbon electrode (GCE) modified with ZIF-8/PANI nanocomposite has been fabricated, and then used for electrochemical sensing of IMZ. Benefitting from the great surface area of ZIF-8 and the good conductivity of PANI, the proposed method exhibited a great electrocatalytic performance towards IMZ reduction in PBS. Furthermore, ZIF-8/PANI nanocomposite shows appreciable recovery results in the practical analysis of vegetable sample. To our best knowledge, there has been no other report on detecting IMZ by the use of ZIF-8/PANI-modified electrochemical sensors.

## Experimental

### Chemicals

Imidaclothiz (99.3%) was purchased from Dr. Ehrenstorfer GmgH (Augsburg, Germany). Methyl parathion (MP), chloramphenicol (CAP), and fenitrothion (FNT) were purchased from Sigma-Aldrich (Shanghai, China). Zn(NO_3_)_2_·6H_2_O and 2-methylimidazole, aniline, ammonium peroxydisulfate (APS), methanol, HCl, NaOH, Na_2_HPO_4_, KH_2_PO_4_, NaCl, KCl, FeCl_3_·6H_2_O, MgCl_2_, NH_4_Cl were purchased from Sinopharm Chemical Reagent Co., Ltd (Shanghai, China). The standard phosphate buffer solutions (0.1 M) with different pH values (from 5.0 to 9.0) were prepared by mixing KH_2_PO_4_ and Na_2_HPO_4_ and adjusting the pH by adding HCl or NaOH.

### Synthesis of ZIF-8 and PANI

First, 2-methylimidazole (5.19 g) and Zn(NO_3_)_2_ (2.346 g) were dissolved, respectively, in 160 mL methanol. These two solutions were mixed under vigorously stirring at ambient temperature. Then, the mixture was stirred for 1 h and the precipitate was collected by centrifugation, followed by washing three times with methanol. Finally, the product was dried in a blast oven at 80 °C overnight, and the ZIF-8 powder was obtained.

PANI synthesis was adapted from a one-pot synthetic procedure reported by Jordan et al. [30]. At first, aniline (0.12 g) was dissolved in a vial containing 4 mL of 1 M hydrochloric acid. The ammonium peroxydisulfate precursor (APS) solution created by dissolving APS (0.29 g) in 4 mL of 1 M hydrochloric acid was then rapidly added to the aniline precursor solution. The solution was shaken for approximately 30 s and then left undisturbed to polymerize for 24 h at room temperature. PANI was then centrifuged and washed several times with distilled water and alcohol to remove excess APS.

### Modification of electrodes

Before modification, the bare GCE surface was polished with Al_2_O_3_ powder down to 0.05 μm particle size and rinsed thoroughly with deionized water and ethanol. To fabricate the ZIF-8/PANI/GCE, the as-synthesized PANI (10 mg) and ZIF-8 (10 mg) were ultrasonically dispersed in 1 mL distilled water for 30 min at first. Next, 5 μL of dispersed PANI solution was dropped directly on the carbon surface of the electrode (precleaned) and dried at room temperature. Then, the obtained PANI/GCE was coated with different volume of ZIF-8 dispersed solution. After the modified electrode was dried, the ZIF-8/PANI/GCE was obtained. For comparison, the ZIF-8/GCE and PANI/GCE were also fabricated for electrochemical investigation.

### Characterization methods and electrochemical measurements

Scanning electron microscopy (SEM) (Zeiss Sigma 300) was adopted for the microscopic morphology characterization of ZIF-8, PANI and ZIF-8/PANI nanocomposite. Nitrogen adsorption–desorption data were obtained using the Micromeritics ASAP 2020 M analyzer, and the crystalline structure of the different materials were characterized by X-ray diffraction (XRD) (Bruker D8 ADVANCE). Also, Fourier transform infrared spectroscopy (FTIR) (Thermo Scientific Nicolet iS5) was employed within KBr slices to identify the presence of function groups in the samples. All electrochemical experiments were conducted at room temperature on CHI-660C electrochemical workstation (Shanghai Chenhua Instrument Co., Ltd., China) by using an electrochemical cell (10 mL) with a standard three-electrode system: the bare or modified glassy carbon electrode as working electrode, a Pt wire counter electrode and an Ag/AgCl reference electrode. Electrochemical studies on the determination of IMZ were performed by using cyclic voltammetry (CV) and square wave voltammetry (SWV) in N_2_-saturated phosphate buffer solution (PBS) (0.1 M, pH = 7), and all error bars show the SD for three independent experiments. Before detection, each sensor was immersed in the solution for several minutes to allow the accumulation of IMZ.

### Real sample preparation

Vegetable sample purchased from local market was first crushed, and then a 30.0 g sample was mixed with 10 g of sodium chloride and 60 mL acetonitrile in a 150 mL beaker. After that, the mixture was homogenized in a blender for 20 min, and 50 mL of supernatant was collected for filtration. Then, the filtrate was dried in nitrogen atmosphere. For electrochemical analysis, the resulting residue was diluted to 10 mL with 0.1 M PBS.

## Results and discussion

### Characterization of ZIF-8/PANI nanocomposite

**Fig. 1:**
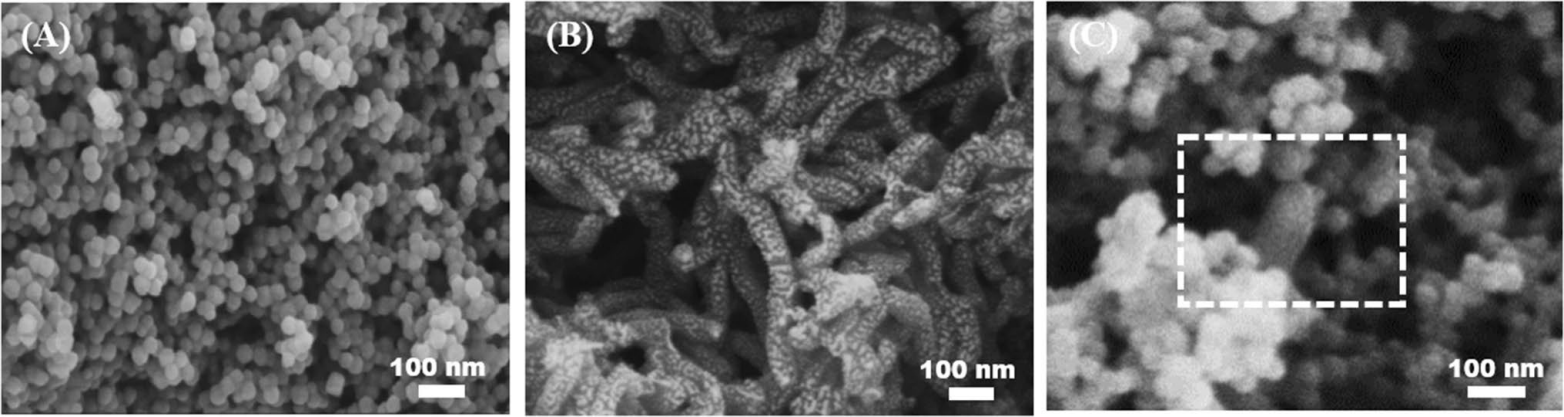
SEM images of ZIF-8 (**A**), PANI (**B**) and ZIF-8/PANI (**C**)

The morphologies of ZIF-8, PANI, and ZIF-8/PANI were investigated by SEM. Figure 1A shows that the ZIF-8 crystals are uniform hexagonal nanoparticles, and the average size is 40 nm approximately. As depicted in Fig. 1B, PANI shows a meshed fibrous structure. Therefore, conductive PANI coated on the surface of GCE can act as bridges among MOF nanoparticles to interconnect ZIF-8. Figure 1C shows that, ZIF-8/PANI nanocomposite can cover the electrode surface uniformly. The isotherm obtained from the N_2_ adsorption–desorption experiment reveals the highly porous texture of ZIF-8 (Fig. S1, Supporting Information), as the BET surface area was 1615 m^2^/g. The XRD patterns (Fig. S2 A, Supporting Information) indicated that ZIF-8 and PANI were prepared successfully and the final composite of ZIF-8/PANI displayed a similar XRD pattern as ZIF-8 with slight differences in peak intensities. The FTIR spectra were performed to analyze the molecular structure of PANI, ZIF-8 and ZIF-8/PANI, and results also showed that ZIF-8 and PANI have been successfully combined (Fig. S2 B, Supporting Information).

### Electrochemical behaviors of IMZ at different modified electrodes

**Fig. 2:**
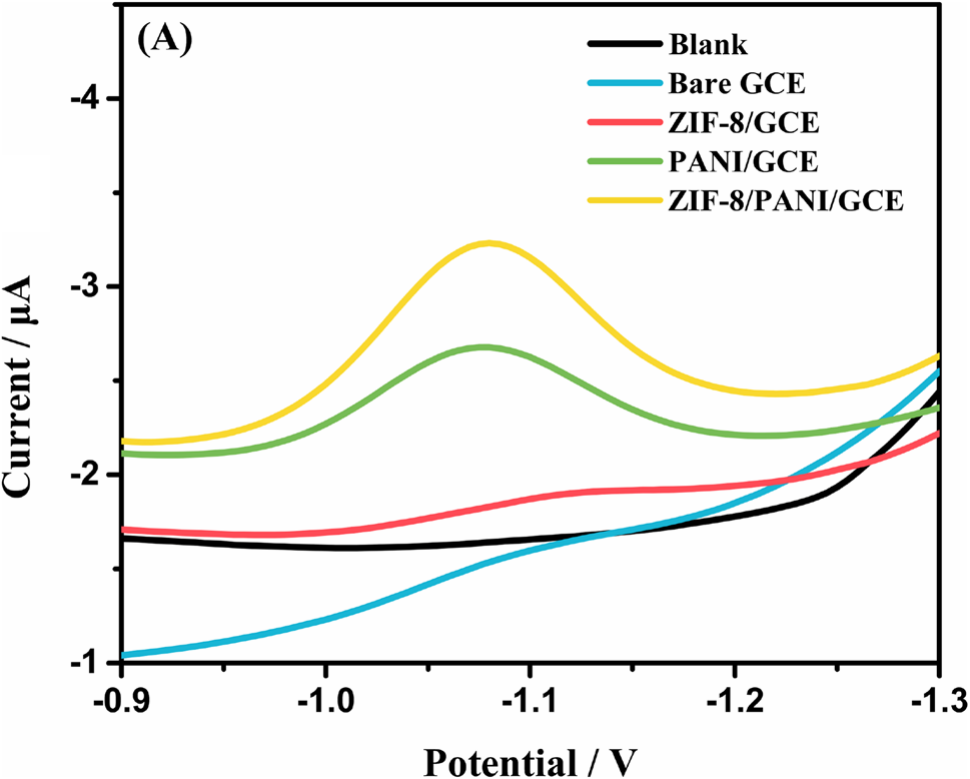
SWV curves for bare GCE, ZIF-8/GCE, PANI/GCE, ZIF-8/PANI/GCE in 0.1 M (pH = 7) PBS containing IMZ (5.0 μM)

IMZ has the electroactive aromatic nitro group, which can present reduction potential between about − 1.3 and − 0.9 V [31]. As shown in Fig. 2, electrochemical behaviors of IMZ on bare GCE, ZIF-8/GCE, PANI/GCE, and ZIF-8/PANI/GCE in PBS were explored by SWV. At first, the negligible peak current of IMZ on bare GCE indicated its little effect in detecting IMZ. Also, due to the poor conductivity of MOFs, no obvious increase of IMZ peak current was observed on ZIF-8/GCE. Then, owing to the great conductivity of PANI, the electrochemical response was improved at PANI/GCE. Significantly, the highest reduction peak current was obtained after modified with ZIF-8/PANI nanocomposite. The increased activity of the ZIF-8/PANI could be attributed to the synergistic effect between ZIF-8 and PANI with large surface area and excellent conductivity. Therefore, the mixing of ZIF-8 and PANI played a key role in improving the electrocatalytic activity of GCE for detection of IMZ. CV curves of different electrodes (Fig. S3, Supporting Information) also revealed the peak associate with aromatic nitro group reduction.

### Optimization of experimental conditions

**Fig. 3:**
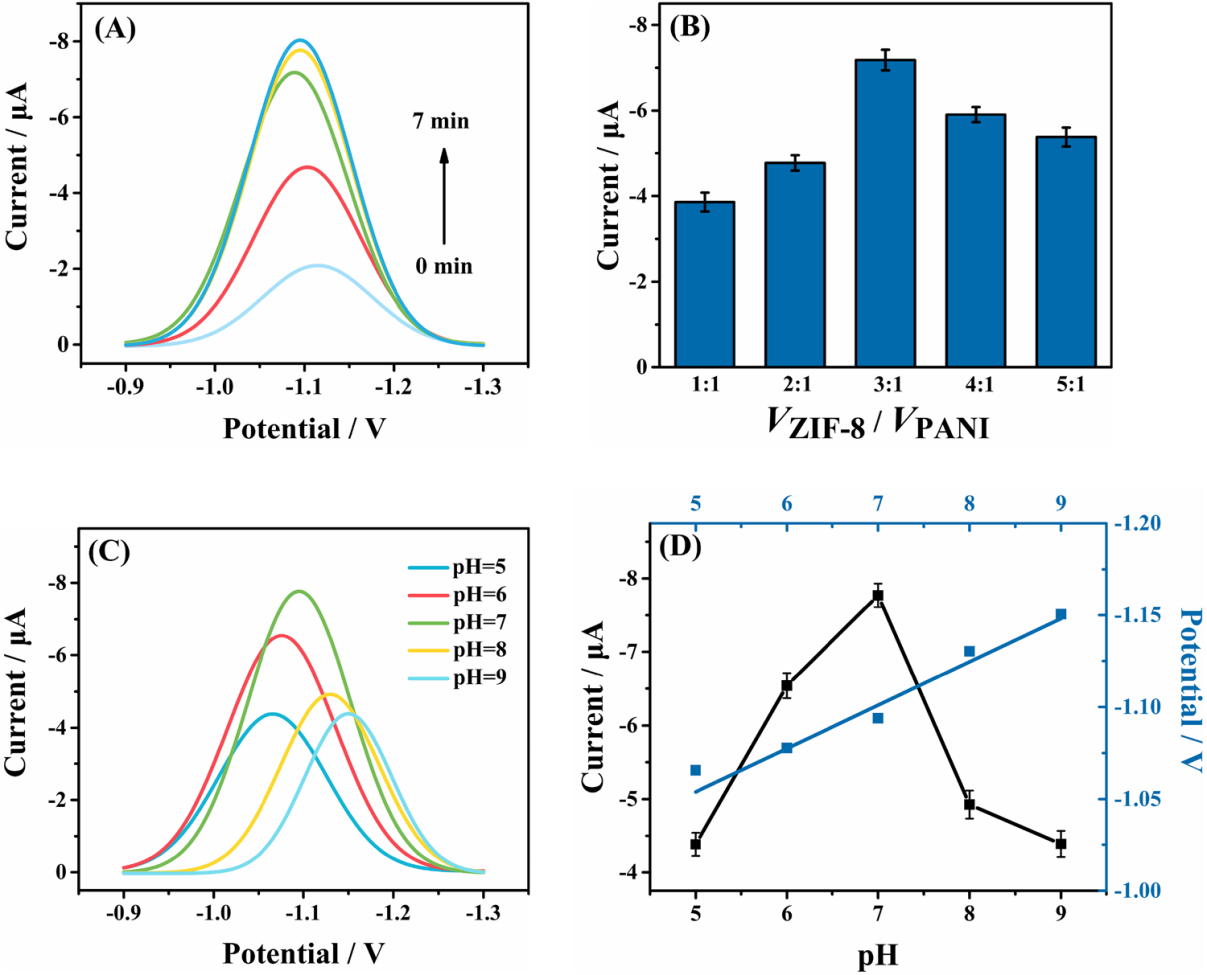
**A** SWV curves (baseline corrected) of 5.0 μM IMZ in 0.1 M PBS on ZIF-8/PANI/GCE with different accumulation time; **B** the volume ration of ZIF-8 and PANI, and **C** pH; **D** effect of pH on the peak current and peak potential

**Scheme 1:**
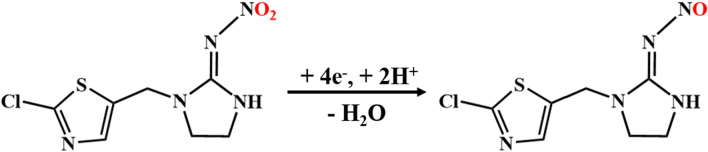
Illustration of electrochemical reduction of IMZ

To obtain higher peak current at the modified electrode, we then investigated the effect of accumulation time on electrochemical performance. As shown in Fig. 3A, the peak current increased gradually with the increase of accumulation time. From 0 to 5 min, the increase in current peak was apparent, whereas little increase was observed when longer accumulation time was used. To meet the demand of rapid and accuracy electrochemical detection, we chose the 5 min for further experiments. The effect of coating amount of composite on the peak current was investigated by changing the volume ratio of PANI and ZIF-8 while the volume of PANI was fixed. In Fig. 3B, the volume ratio of ZIF-8 and PANI exerted apparent effect on the electrochemical response to IMZ, and showing the greatest current peak at the ratio of 3:1. The Ph has a deep influence on the electrocatalysis of IMZ on the surface of the ZIF-8/PANI/ GCE by affecting the peak current and peak potential. As shown in Fig. 3C, the electrochemical behaviors of IMZ in PBS over the Ph range of 5–8 were investigated. The reduction peak current of IMZ increased slightly with the increasing Ph from 5 to 7 and then decreased, so the Ph = 7 was selected as the optimal condition in this work. Furthermore, Fig. 3D shows that the peak potentials (Ep) of IMZ shifted negatively with the increasing Ph, and the liner regression equation was *Ep* (*V*) = − 0.025 × *Ph*−0.928 (*R*^2^ = 0.971). The slope was close to the theoretical value (30 Mv/Ph) according to Nernst equation, indicating that the ratio of protons and electrons involved in the electrochemical reaction was 0.5. Thus, the conjectured reduction mechanism of IMZ on ZIF-8/PANI/GCE is given in Scheme 1, the nitro group of IMZ was reduced to the corresponding nitroso group by gaining 4e^−1^ and 2H^+^ during the reduction process.

Parameters of SWV method including frequency, amplitude, and step potential were optimized in the range of 10–60 Hz, 20–70 Mv, and 5–25 Mv, respectively (Fig. S4, Supporting Information). The recorded results revealed that, on increasing the frequency, the peak current increased steadily but got decreased after 50 Hz. Therefore, the 50 Hz should be set as the optimal frequency (Fig. S4 A, Supporting Information). Similarly, for the amplitude and the step potential, 60 mV and 20 mV were found to be the optimal values (Fig. S4 B and Fig. S4 C, Supporting Information).

### Determination of IMZ

**Fig. 4:**
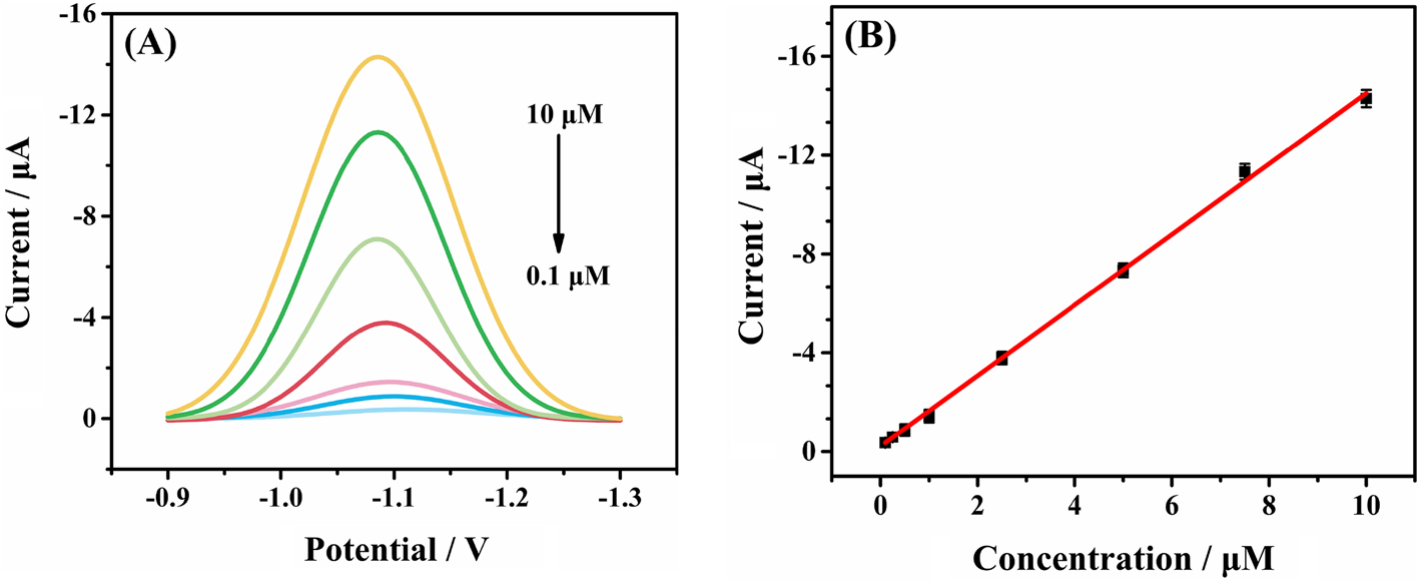
**A** SWV curves (baseline corrected) of different concentrations of IMZ (from bottom to top: 0.1, 0.25,0.5, 1.0, 2.5, 5.0, 7.5, 10.0 μM) under the optimized conditions; **B** the relationship between peak currents with concentrations

SWV was applied to evaluate the sensitivity of this sensing system. Generally, a good linear relationship and a low limit of detection (LOD) are considered to be the key parameters in evaluating an electrochemical sensor. The prepared ZIF-8/PANI/GCE was used to detect IMZ in 0.1 M PBS under the optimized conditions. As seen from Fig. 4A, the measured current response increased successively along with the concentration of IMZ in the range of 0.1–10 μM, and a good linear relationship between the peak current and IMZ concentration was achieved with the equation of *I* (− *μA*) = − 1.43 × *C* (*μM*)−0.22 (*R*^2^ = 0.998) as shown in Fig. 4B. The LOD of 25 nM (S/N = 3) was achieved. Our experimental results showed that the electrochemical senor of ZIF-8/PANI/GCE for IMZ detection exhibited not only device simplicity but also good sensitivity.

The LOD found in this paper was also compared with the previously reported methods for determination of IMZ and other neonicotinoids. As shown in Table S1 (Supporting Information), the LOD of the immunoassay and UPLC–MS/MS are lower than electrochemical methods, but these methods usually need high instrument cost and sophisticated sample preparation processes. Many electrochemical sensors have been used in neonicotinoids detection, while no IMZ sensor has been fabricated before this work. The comparative results demonstrated that this performed method exhibits a low detection limit and even similar as the other electrochemical sensors.

### Selectivity, reproducibility, and stability

The selectivity of ZIF-8/PANI/GCE toward IMZ was evaluated under the presence of 10 times concentration of potential interferences, including Na^+^, K^+^, Fe^3+^, Mg^2+^, NH_4_^+^, and several organic compounds, such as methyl parathion (MP), chloramphenicol (CAP) and fenitrothion (FNT), which also contain aromatic nitro. It is clear that these potential interferences had negligible influences on the current responses of IMZ (Fig. S5, Supporting Information). The variations range of recovery current was from − 1.41% to − 7.17%, suggesting that the proposed method was relatively selective for the detection of IMZ. To investigate the reproducibility of the electrode, five parallel tests using ZIF-8/PANI/GCE were measured in 2.5 μM of IMZ. From the results shown in Fig. S6 A (Supporting Information), the relative standard deviation (RSD) was calculated to be 3.09%, revealing the excellent reproducibility of the as-prepared electrodes. Besides, the modified electrodes were stored at 4 °C and intermittently measured the response currents of 2.5 μM IMZ every 3 days over the period of 15 days (Fig. S6 B, Supporting Information). The results showed that the peak currents remained about 92.99% of their initial value, reflecting a good stability for the electrochemical detection.

### Real sample determination

To verify the reliability of the developed method for IMZ determination in real sample, the developed method was applied to detect IMZ in vegetable sample. To determine insecticide concentration in the real sample, different amounts of IMZ were added individually in real sample. The analytical results of the ZIF-8/PANI/GCE in the presence of IMZ in real sample are summarized in Table 1. The recovery rates of IMZ in real sample were between 91.10% and 104.25%, and the RSD was 4.26%, indicating that the method could detect IMZ in real sample.

**Table 1:** Determination of IMZ in real sample on ZIF-8/PANI/GCE

Added/μM	Recovery, %	RSD, %
0.25	91.10–95.46	2.06
1.00	95.76–104.50	4.26
2.00	98.05–104.25	2.60

## Conclusions

In summary, this work developed and validated an electroanalytical method based on ZIF-8/PANI nanocomposite for sensitive IMZ determination. Taking advantage of the synergistic effect between ZIF-8 and PANI, the prepared nanocomposite exhibited large activity surface area and excellent conductivity. The fabricated sensor showed a superior performance for electrochemical detection of IMZ with a good linear response towards IMZ in a range of 0.1–10 μM and a low LOD value of 25 nM. With the great advantages including high sensitivity and good stability, this MOFs-based sensor provided an excellent performance towards the determination IMZ in vegetable sample, holding great potential for IMZ residues analysis.

### Supplementary Information

Below is the link to the electronic supplementary material.

Supplementary file1 (PDF 488 KB)
