# Supplementary material for: Zeolitic imidazolate framework-8/polyaniline nanocomposite-based electrochemical sensor for sensitive detection of imidaclothiz
Source: Anal Sci. 2023 Oct 25;39(11):1857–63. doi: 10.2116/analsci.21P063 (PMC10598144; doi:10.2116/analsci.21P063)
Supplement: Supplementary file 1 — Supplementary file1 (PDF 488 KB) [file 44211_2023_418_MOESM1_ESM.pdf]

Supporting Information

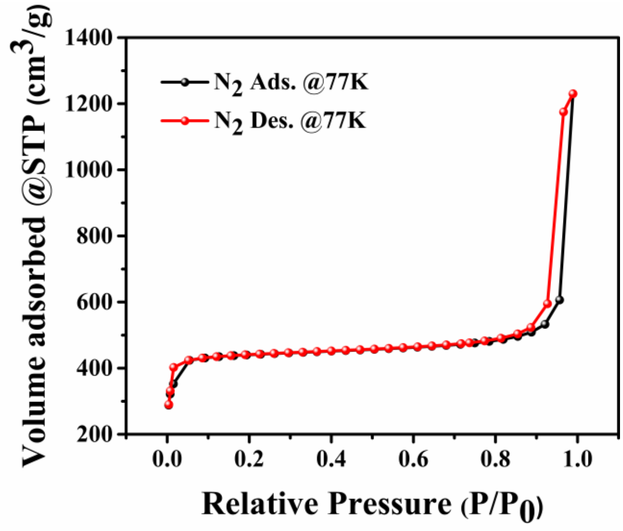

Fig. S1 N_2 adsorption-desorption isotherm for ZIF-8 at 77 K.

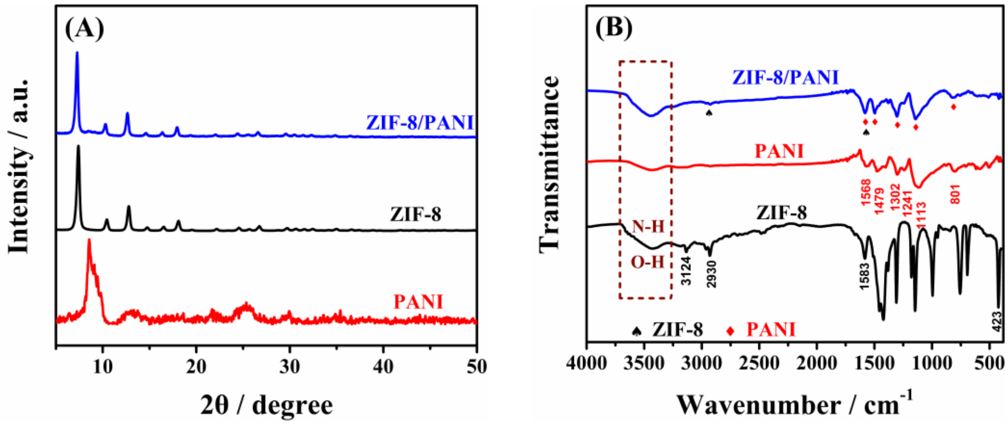

Fig. S2 (A) XRD patterns and (B) FTIR spectra of the ZIF-8, PANI, and ZIF-8/PANI.

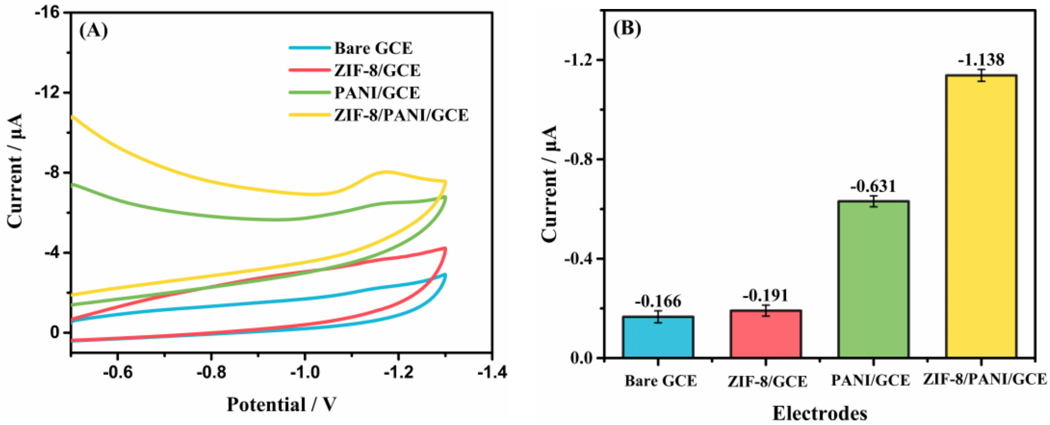

Fig. S3 (A) CV curves for bare GCE, ZIF-8/GCE, PANI/GCE, ZIF-8/PANI/GCE in 0.1 M (pH = 7) PBS containing IMZ (5.0 μM), scan rate: 50 mV/s; (B) peak current of SWV curves (baseline-corrected) versus different electrodes.

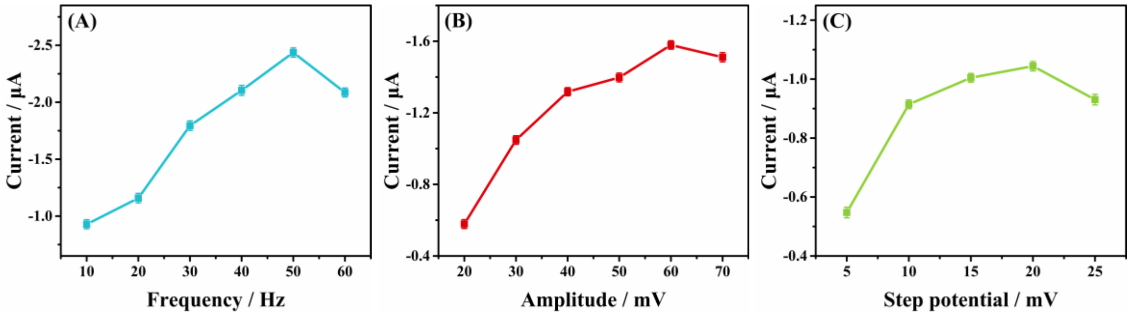

Fig. S4 Optimization of the SWV technique parameters in 0.1M PBS (pH =7) containing IMZ (5.0 μM), corresponding reduction peak current after baseline correction: (A) frequency (10-60 Hz), (B) amplitude (20-70 mV), (C) step potential (5-25 mV).

Table S1 Comparison of the proposed method with other methods for the determination of imidaclothiz and other neonicotinoids.

Determination methods	Analyte	LOD	Liner range	Ref.
MSFIA[a]	Imidaclothiz	1.87 ng mL^{-1}	1.87 - 66.0 ng/mL	1
		(7.22 nM)	(7.22 - 252 nM)	
UPLC-MS/MS[b]	Imidaclothiz	0.04 μg L^{-1}	0.1 - 200 μg/L	2
		(0.15 nM)	(0.38 – 764 nM)	
Electrochemical sensor	Imidacloprid	8.92 μM	5 - 165 μM	3
	Clothianidin	4.72 μM	10 - 80 μM	
	Thiamethoxam	7.45 μM	10 - 70 μM	
Electrochemical sensor	Imidacloprid	7.9 μM	10 - 200 μM	4
	Thiamethoxam	8.3 μM	10 - 200 μM	
Electrochemical sensor	Imidacloprid	0.026 μM	0.5 - 60 μM	5
	Thiamethoxam	0.062 μM	1 - 60 μM	
	Dinotefuran	0.01 μM	0.5 - 60 μM	
Electrochemical sensor	Thiamethoxam	4.9 nM	0.01 - 420 μM	6
Electrochemical sensor	Imidaclothiz	0.025 μM	0.1 - 10 μM	This work

[a] Magnetic-separation fluorescence immunoassay

[b] Ultra-high performance liquid chromatography coupled with tandem mass spectrometry

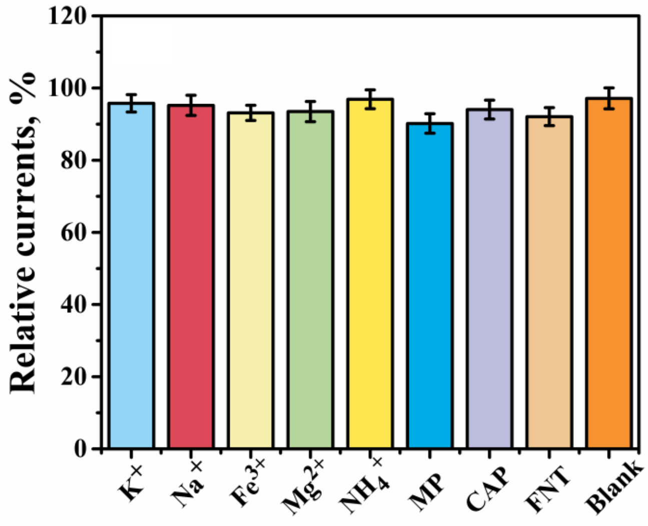

Fig. S5 The bar diagram of ZIF-8/PANI/GCE at presence of interfering compounds such as K^+, Na^+, Fe^{3+}, Mg^{2+}, NH_4^+, methyl parathion(MP), chloramphenicol (CAP) and fenitrothion (FNT).

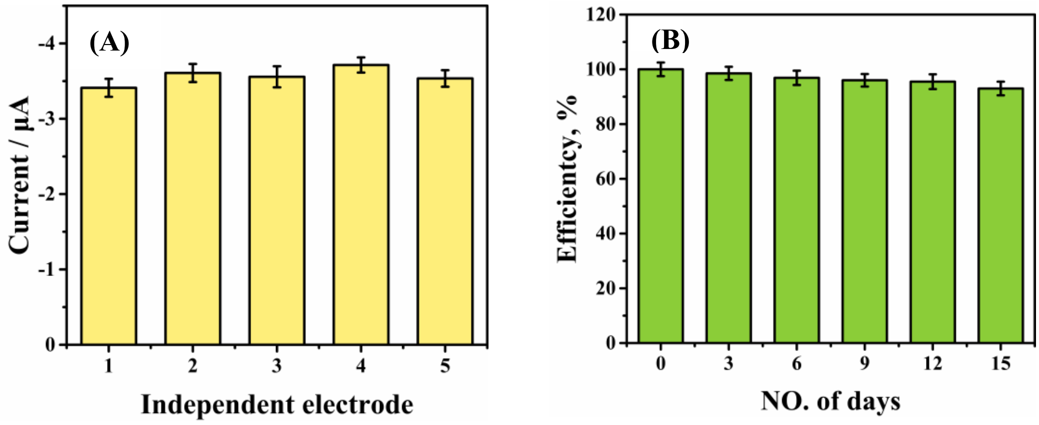

Fig. S6 (A) The reproducibility of the sensor over the 5 different ZIF-8/PANI/GCE. (B) the histogram of ZIF-8/PANI/GCE stability at IMZ detection for 15 days.

References

1. Y. Ding, X. D. Hua, M. Du, Q. Yang, L. N. Hou, L. M. Wang, F. Q. Liu, G. Gonzalez-Sapienza and M. H. Wang, *Anal. Chem.*, **2018**, *90*, 13996.

2. Q. Zhang, X. M. Wang, Z. Li, H. B. Jin, Z. B. Lu, C. Yu, Y. F. Huang and M. R. Zhao, *Environ. Pollut.*, **2018**, *240*, 647.

3. A. E. F. Oliveira, G. B. Bettio and A. C. Pereira, *Electroanal*, **2018**, *30*, 1918.

4. V. Urbanova, A. Bakandritsos, P. Jakubec, T. Szambo and R. Zboril, *Biosensors & Bioelectronics*, **2017**, *89*, 532.

5. Q. Wang, H. Zhangsun, Y. Zhao, Y. Zhuang, Z. Xu, T. Bu, R. Li and L. Wang, *J. Hazard. Mater.*, **2021**, *411*, 125122.

6. J. Ganesamurthi, M. Keerthi, S. M. Chen and R. Shanmugam, *Ecotox Environ Safe*, **2020**, *189*.
